# Contamination of filtering face piece 3 masks with SARS-COV-2 during endotracheal intubation

**DOI:** 10.1017/ice.2020.269

**Published:** 2020-06-03

**Authors:** Alice Bone, Elizabeth Barton, Shirley Hoskins, Abigail Holborow, Claire Johnston, Ian Blyth, Jonathan Evans, Brendan Healy

**Affiliations:** 1 Public Health Wales Microbiology, Swansea, Wales, United Kingdom; 2 Critical Care, Swansea Bay University Health Board, Swansea, Wales, United Kingdom; 3 Emergency Care and Hospital Operations, Swansea Bay University Health Board, Swansea, Wales, United Kingdom; 4 Virology, Public Health Wales Microbiology, Cardiff, Wales, United Kingdom; 5 Microbiology and Infectious Diseases, Public Health Wales Microbiology, Swansea, Wales, United Kingdom

*To the Editor*—In response to the article published on contamination of personal protective equipment (PPE) by severe acute respiratory syndrome coronavirus 2 (SARS-CoV-2),^1^ we describe a small study investigating contamination and decontamination of filtering face piece 3 (FFP3) masks after exposure to SARS-CoV-2 in a variety of settings, including routine patient care and during endotracheal intubation.

In response to the COVID-19 global pandemic, countries have implemented strategies to limit the spread of disease. In the United Kingdom, healthcare workers (HCWs) are advised to wear PPE in line with Public Health England (PHE) guidance. Maintaining PPE supplies has become a priority for healthcare systems around the world.

Human transmission of SARS-CoV-2 is thought to occur predominately via close contact through droplets produced from the respiratory tract or fomites. In hospitals, airborne transmission is possible in specific circumstances during aerosol-generating procedures (AGPs) when respiratory secretions are exposed to high pressure. PHE recommends an FFP3 mask, eye protection, long-sleeved gown, and gloves for personnel at risk of exposure to AGPs. Endotracheal intubation is classed as an AGP.^2^

In this study, we sampled 8 FFP3 masks (Aura, 3M, St Paul, MN) after exposure to COVID-19–positive patients. Two masks were worn by HCWs during routine patient care on a normal shift on a ward of confirmed COVID-19 patients.

The remaining 6 masks were exposed during endotracheal intubation of 2 COVID-19–positive patients. For each intubation, 3 exposed masks were analyzed. One mask was held immediately to the left of the patient at shoulder height, ~40 cm above the patient’s sternal notch with the outer filter pointing toward the patient (positive control). One mask was worn by the clinician carrying out the intubation, and the other was worn by the assisting clinician.

Masks were sampled before and after decontamination as follows: 1 dry swab was used to swipe the outside surface including the outer filter and 1 dry swab was used to swipe the inside surface. A sample was then taken from the front of the mask using a hole punch for uniformity. The masks were decontaminated on the Antigermix AE1 Probe Disinfector using a 40-second cycle of ultraviolet germicidal irradiation (UVGI).

In total, 4 swabs and 2 samples of material were tested for each mask, yielding a total of 48 data points. Samples were analyzed by real-time PCR using an in-house assay. For positive samples, the cycle threshold (CT) value (ie, the number of PCR cycles required before the result flags as positive) was recorded. A lower CT value is generally associated with a higher viral load.

Low levels of SARS-CoV-2 were detected from 2 of the 6 samples taken from 2 masks used as positive controls (Table 1). Both samples were taken from masks held to the side of a patient during endotracheal intubation (1 positive mask from each patient). Both masks tested negative after decontamination with UVGI.

**Table 1.** Contamination of Masks With SARS-COV-2

Mask No.	SARS-COV-2 Detected	
	Before Decontamination	After Decontamination
Mask 1		
Outside swab (CT value)	Yes (39.9)	No
Inside swab	No	No
Sample	No	No
Mask 2		
Outside swab	No	No
Inside swab	No	No
Sample	No	No
Mask 3		
Outside swab	No	No
Inside swab	No	No
Sample	No	No
Mask 4		
Outside swab	No	No
Inside swab (CT value)	Yes (37.82)	No
Sample	No	No
Mask 5		
Outside swab	No	No
Inside swab	No	No
Sample	No	No
Mask 6		
Outside swab	No	No
Inside swab	No	No
Sample	No	No
Mask 7		
Outside swab	No	No
Inside swab	No	No
Sample	No	No
Mask 8		
Outside swab	No	No
Inside swab	No	No
Sample	No	No

Note. CT, cycle threshold.

SARS-COV-2 was not detected on the masks of HCWs delivering routine care (ie, no AGPs performed) to COVID-19–positive patients (Table 1). SARS-COV-2 was not detected from any samples taken after decontamination using UVGI (Table 1).

No contamination of face masks worn by HCWs during care for known COVID-19 patients (whether worn during intubation or for routine patient care on a ward) was detected. Full face visors were worn by all staff members, which may have blocked contamination of the underlying mask.

Previous studies have investigated contamination of PPE with SARS-COV-2. No contamination was found on N95 masks, goggles, or shoes of exposed HCWs.^1^ Notably, no AGPs were performed in this study. In a study published in Singapore, extensive environmental contamination with SARS-COV-2 was detected in the healthcare setting, but only 1 sample of 10 taken from PPE was positive (the front of 1 HCW’s shoe).^3^

Another study in which masks were artificially contaminated with a high-concentration SARS-CoV-2 solution has brought the effectiveness of UVGI in decontamination into question.^4^ The authors state that it is “hard to imagine a scenario where HCWs would face this degree of mask inoculum” and that their data may inadvertently underestimate decontamination efficacy.^4^ Our study supports this assertion because contamination was not demonstrated during routine care and intubation.

Our study was limited by the small number of masks sampled and the relatively high CT values of the intubated patients (ie, 32 and 36). AGPs in patients with lower CT values and hence higher viral loads may result in greater contamination.

Despite FFP3 masks being marketed as single use, reuse has been suggested by external bodies as a contingency capacity strategy in a crisis situation. Despite the small sample size, it is reassuring that SARS-COV-2 was not detected on any masks worn by HCWs and that no virus was detected after decontamination with UVGI. Importantly, filtration and fit were not retested, and decontamination methods (including UVGI) may affect mask integrity. More evidence is required prior to routine adoption of any such process.

In summary, we have demonstrated that, when worn behind full-face visors, contamination of masks is uncommon during intubation and during extended periods of routine care of COVID-19–positive patients. Limited contamination of exposed face masks was documented during intubation, an aerosol-generating procedure. No virus was detectable after a 40-second UVGI decontamination process.
